# Latent profile analysis for health-related quality of life, sleep quality, morning and evening type, and internet addiction among medical students

**DOI:** 10.1038/s41598-023-38302-7

**Published:** 2023-07-12

**Authors:** Ling Yu, Yifan Wu, Chaowei Guo, Qiao Qiao, Xue Wang, Shuang Zang

**Affiliations:** 1 Department of Community Nursing, School of Nursing, China Medical University, No.77 Puhe Road, Shenyang North New Area, Shenyang, 110122 Liaoning Province China; 2 Phase I Clinical Trails Center, The First Hospital of China Medical University, No.155 Nanjing Bei Street, Heping District, Shenyang, 110001 Liaoning Province China; 3 School of Nursing, Jilin University, 965 Xinjiang Street, Changchun, 130021 Jilin Province China; 4 Department of Radiation Oncology, The First Hospital of China Medical University, No.155 Nanjing North Street, Heping District, Shenyang, 110001 Liaoning Province China

**Keywords:** Health care, Risk factors

## Abstract

Health-related quality of life, sleep quality, morning and evening types, and internet addiction are of significant importance to the development of medical students, yet they have rarely been studied. Taking this into consideration, the study aimed to confirm latent profiles in health-related quality of life, sleep quality, morning and evening types, and internet addiction in medical students and investigate the characteristics of participants in each profile to provide suggestions for students’ health. This was an observational cross-sectional study including 1221 medical student subjects at China Medical University in 2019. Multiple correspondence analysis was the initial step to verify the correspondence, dispersion, and approximation of variable categories. Latent profile analysis was used to identify the multiple correspondences between the levels of variables. Three profiles were found, including: (1) The Low sleep quality profile was characterized by the lowest sleep quality among the three existing profiles. (2) The High health-related quality of life and Low internet addiction profile was characterized by the highest level of health-related quality of life but the lowest level of internet addiction. (3) The Low health-related quality of life and High internet addiction profile was characterized by the highest standardized values of internet addiction but the lowest standardized values of health-related quality of life. This study had important implications for improving student health and supported the medical universities and hospitals in implementing targeted policies based on distinctive student characteristics.

## Introduction

Sleep problems are very common in the general population, and medical students are a vulnerable group that is prone to poor sleep quality^1^. According to a comprehensive meta-analysis of 57 studies with 25,735 medical students, the pooled prevalence of poor sleep quality was 52.7%^2^. Another study found that in the United States, medical students' sleep quality was significantly worse than a healthy adult sample^3^. Similarly, a cross-sectional study in Etawah college also reported that medical students had worse sleep quality than nonmedical students^4^. As a key component of personal health in medical education, the sleep quality of students cannot be ignored.

The sleep quality of medical students at the university was affected by living habits, such as electronic use before bed and sleep duration^5^. As sleep-interfering processes theory indicates, sleep problems arise when the brain is highly active before bedtime, such as rumination, worrying, planning, analyzing, problem-solving, and difficulty controlling one’s thoughts^6^. If the person engages in sleep incompatible behaviors in bed (browsing the internet before bedtime, staying in bed late, poor sleep habits, etc.), sleep disturbances are likely to occur^6^. For example, a study of young adults aged 19–32 showed that using social media within 30 min of bedtime was independently associated with sleep disturbance^7^. Another study found that strict parental rules about internet and smartphone use before sleep might prevent negative consequences of social media use on bedtime and sleep quality^8^.

However, the living habits of young people transitioning from secondary education to university education have undergone profound changes, with the main manifestation being that many students have a tendency to sleep late or a nighttime preference, which is particularly prominent among medical students^9^. A study has shown that medical students can be obliged to change their morning and evening type because of academic commitments, such as working night shifts during internships, which can lead to long-term sleep deprivation and cumulative sleep deficits^10^. In addition, there is a high prevalence of internet addiction among medical undergraduate students^11,12^. The researchers found that although some medical students used the internet for academic searches, the majority of them used it for entertainment^13^. A study of 337 medical students in Tumakuru also confirmed that medical students had a significantly higher prevalence of internet addiction than the general population^14^. More than half of medical students lost sleep due to late-night internet use^15^.

The above findings are concerning because the evening type and internet addiction can have compounding effects, such as causing medical students to be absent from daytime activities, with negative effects on academic performance and health-related quality of life^16,17^. As a result, sleep quality and health-related quality of life interact with each other and progress negatively, creating a chronic cycle.

Health-related quality of life refers to a subjective rating of the impact of an individual’s health on his or her overall well-being^18^. Currently, students in the health professions have a worse health-related quality of life and a relatively low awareness of issues related to quality of life, compared to the general population^19–21^. The poor health-related life quality of medical students remains a significant issue for the profession to address.

Therefore, this study focused on sleep quality, morning and evening patterns, internet addiction, and health-related quality of life to identify and manage the health problems of medical students early. A growing body of research has examined the associations between health-related quality of life, sleep quality, morning and evening type, and internet addiction in the general population, including studies on medical students^22–25^. However, few studies have used a person-centered approach (i.e., the interest is finding heterogeneous groups of individuals) to explain the relationship between the responses to the observed set of categorical variables. These studies often treat the group of medical students as a single distribution, ignoring the measurable differences among individuals within the group. The use of data-based, person-centered classification is crucial to ensure the healthy growth of students in their lives and to develop targeted primary prevention measures based on profile characteristics^26,27^.

We examine the different health-related quality of life, sleep quality, morning and evening type, and internet addiction clusters by latent profile analysis (LPA), using data from a cross-sectional survey of medical students at China Medical University. Identifying these elements and potential risk categories among medical students will improve their health and formulate policies in a way that does not affect their performance.

## Method

### Sample

This study was a cross-sectional study. A total of 1224 medical students participated in the survey. We compiled and collected the questionnaires through the Questionnaire Star Platform (Changsha Ranxing Information Technology Co., Changsha, China) to investigate the demographic data and health-related quality of life, sleep quality, morning and evening type, and internet addiction of medical students. After removing irregular responses, the study comprised 1221 medical students. This study was approved by the Ethics Committees of China Medical University (No. CMU1210400026). The research was performed in accordance with the Declaration of Helsinki.

### Measures

Sociodemographic data included sex and age. The study variables also included health-related quality of life, sleep quality, morning and evening type, and internet addiction of medical students.

#### Health promoting lifestyle profile II (HPLP II)

The HPLP II was used to measure health-related quality of life^28^. The HPLP II consists of 52 Likert scale items, with a higher score indicating a higher level of health-related quality of life. HPLP-II contains 6 domains: interpersonal relations, spiritual growth, nutrition, stress management, health responsibility, and physical activity. Items are rated using a four-point rating scale, with 1 meaning “never,” 2 meaning “sometimes,” 3 meaning “very often” and 4 meaning “routine.” Total HPLP II scores range from 52 to 208. A score of 52–104 indicates poor marked as “unhealthy”, 105–156 indicates moderate marked as “moderate”, and 157–208 indicates good marked as “health”. The Cronbach’s alpha in this sample for HPLP II is 0.974.

#### Pittsburgh sleep quality index (PSQI)

Students completed the PSQI to measure sleep quality^29,30^. The scale consists of 19 questions evaluating the following seven dimensions: fall asleep time, sleep time, sleep efficiency, sleep disorders, sleep drugs, daytime dysfunction and self-rated sleep quality. The score range for the 16 items is 0–3 points. Item 1 refers to bedtime, item 3 refers to wake-up time, and sleep efficiency is calculated based on items 1, 3, and 4, with a score range of 0–3. The PSQI ranges from 0 to 21, which assesses sleep quality for the past month^31^. The PSQI indicates good sleep quality, with 0–5 points marked as “very good”*,* 6–10 points denoted as “better”, 11–15 points indicated as “general”, and “poor” sleep quality referring to 16–21 points^32^. The PSQI has been validated in college students and has high reliability^6,30^.

#### Morningness-eveningness questionnaire (MEQ)

The MEQ is a self-assessed questionnaire consisting of 19 items that assesses an individual’s chronotype^33^. The total score ranges from 16 to 86 points, consisting of 11 items with a scoring range of 1–4 points, two items with scores of 0, 2, 4, or 6 points, one item with scores of 0, 2, 3, or 5 points, and five items with a scoring range of 1–5 points. This questionnaire establishes three behavioral categories: evening types (late bedtimes and late wake-up, score = 16–41) marked as “evening”, intermediate types (regular type, score = 42–58) marked as “intermediate”, and morning types (early to bed and early to rise, score = 59–86) marked as “morning”. The MEQ has been used in many studies and has demonstrated good measurement performance among college students^34,35^.

#### Internet addiction test

The Internet Addiction Test measures the level of internet addiction based on 20 items using a five-point Likert scale^36^. Each question is scored by 1 meaning “hardly ever,” 2 meaning “occasionally,” 3 meaning “sometimes,” 4 meaning “very often,” and 5 meaning “always.” Scores over 50 indicate occasional or frequent internet addictive behavior. A higher score indicates a higher level of internet addiction. The Cronbach’s alpha in this sample for the internet addiction scale is 0.935.

### Data analysis

Data analyses were performed using the SPSS Statistics Version 23.0 (IBM Corp., Armonk, NY, USA) and EmpowerStats (X&Y Solutions, Inc., Boston, MA, USA). LPA was performed using Mplus Version 7.0 (Muthén and Muthén, Los Angeles, CA, USA). The Shapiro–Wilk test revealed the nonnormal distribution of the data. Comparisons of distributions between three groups were made by the Kruskal–Wallis test (including Dunn-Bonferroni post hoc correction). All tests were two-sided, and statistical significance was set at *P* < 0.05.

LPA was conducted to examine the latent profiles of health-related quality of life, sleep quality, morning and evening type, and internet addiction among medical students. Until the fitness measures reached their ideal levels, models were estimated by gradually increasing the number of profiles from the start (2 profile) to the final model. A log-likelihood test was utilized for model fitting, and the ensuing metrics were commonly employed to signify the aptness: the Akaike information criterion (AIC), the Bayesian information criterion (BIC), and the sample size adjusted Bayesian information criterion (aBIC), with a smaller value indicating better model fitness^37^. In LPA, entropy values are frequently computed to assess the precision of classification, on a scale of 0 to 1, with a predilection for higher values. In addition, *P* was calculated by the Lo-Mendell-Rubin test, and Vuong-Lo-Mendell-Rubin are crucial metrics for determining whether the model best suits the data^37^. If *P* is less than 0.05, it means that the model fits the data significantly superior to the antecedent model^38^.

Multiple correspondence analysis (MCA) was used to identify the most important relationships among variables in the dataset by a graphical representation of the similarity between the given observations (Euclidian distance). In a preliminary step, MCA was used to confirm correspondence, dispersion, and approximation in variable categories. The exploratory method provided the concepts of the index variables and the number of latent profiles through the principal plane. The inertia value and Cronbach’s alpha were used to define the number of dimensions to retain in the final MCA^39^. The results of the first two MCA dimensions were plotted to illustrate relationships between variables as MCA principal dimensions. According to LPA, respondents are assigned to the cluster with the highest attribution probability based on their responses to observed variables. To find the various correspondences between the levels of the categorical variables, a multivariate analysis was used for categorical variables.

### Patient and public involvement

The design of the study aimed to address questions about health-related quality of life, sleep quality, morning and evening type, and internet addiction in medical students. In the formative research stage, we seek input from participants in research design and behavior. All students participated voluntarily and were not remunerated. Although there was no specific public involvement in the development of the research question and outcomes, any student, as a participant in the survey, can obtain contact information to keep up-to-date research results. The preliminary findings were then tested and analyzed in depth before the current study.

### Ethics approval and consent to participate

The study was approved by the institution’s ethics committee (No. CMU1210400026). Written informed consent was obtained from participants included in the study. Any personal information obtained in this study remained confidential. The research was performed in accordance with the Declaration of Helsinki.

## Results

### Participant characteristics

Most of the students (n = 736; 60.28%) in the study were aged 20 years old. The proportion of female students was higher than that of males, and there were 807 females and 414 males in this study. Significant differences were observed between males and females in sleep quality and morning and evening type (*P* < 0.05). There were no significant differences between males and females in health-related quality of life and internet addiction. There was no difference between health-related quality of life, sleep quality, internet addiction, and morning and evening type in different age groups (*P* > 0.05). Details are presented in Table 1.

**Table 1:** Differences in health-related quality of life, sleep quality, morning and evening type, and internet addiction scores by sociodemographic characteristics (n = 1221).

Variables	n (%)	Health-related quality of life		Sleep quality		Morning and evening type		Internet addiction	
		Median (IQR)	*p* value	Median (IQR)	*p* value	Median (IQR)	*p* value	Median (IQR)	*p* value
Sex									
Male	414 (33.90%)	144.00 (126.00, 156.00)	0.522	5.00 (3.00, 7.00)	0.018	47.00 (43.00, 51.00)	< 0.001	47.00 (38.00, 60.00)	0.152
Female	807 (65.84%)	145.00 (129.00, 156.00)		5.00 (3.00, 8.00)		49.00 (45.00, 51.00)		48.00 (40.00, 57.00)	
Age (years)									
≤ 19	178 (14.58%)	145.00 (129.00, 156.00)	0.399	5.00 (3.00, 8.00)	0.136	49.00 (45.00, 52.00)	0.055	46.00 (38.75, 55.00)	0.250
20	736 (60.28%)	145.00 (129.00, 156.00)		5.00 (3.00, 7.00)		48.00 (44.25, 51.00)		48.00 (40.00, 58.00)	
21	264 (21.62%)	143.50 (128.00, 156.00)		6.00 (4.00, 8.00)		48.00 (44.00, 51.00)		49.00 (40.00, 59.00)	
≥ 22	43 (3.52%)	111.00 (138.00, 160.00)		5.00 (3.00, 8.00)		48.00 (44.00, 51.00)		47.00 (41.00, 55.00)	

### MCA

**Figure 1:**
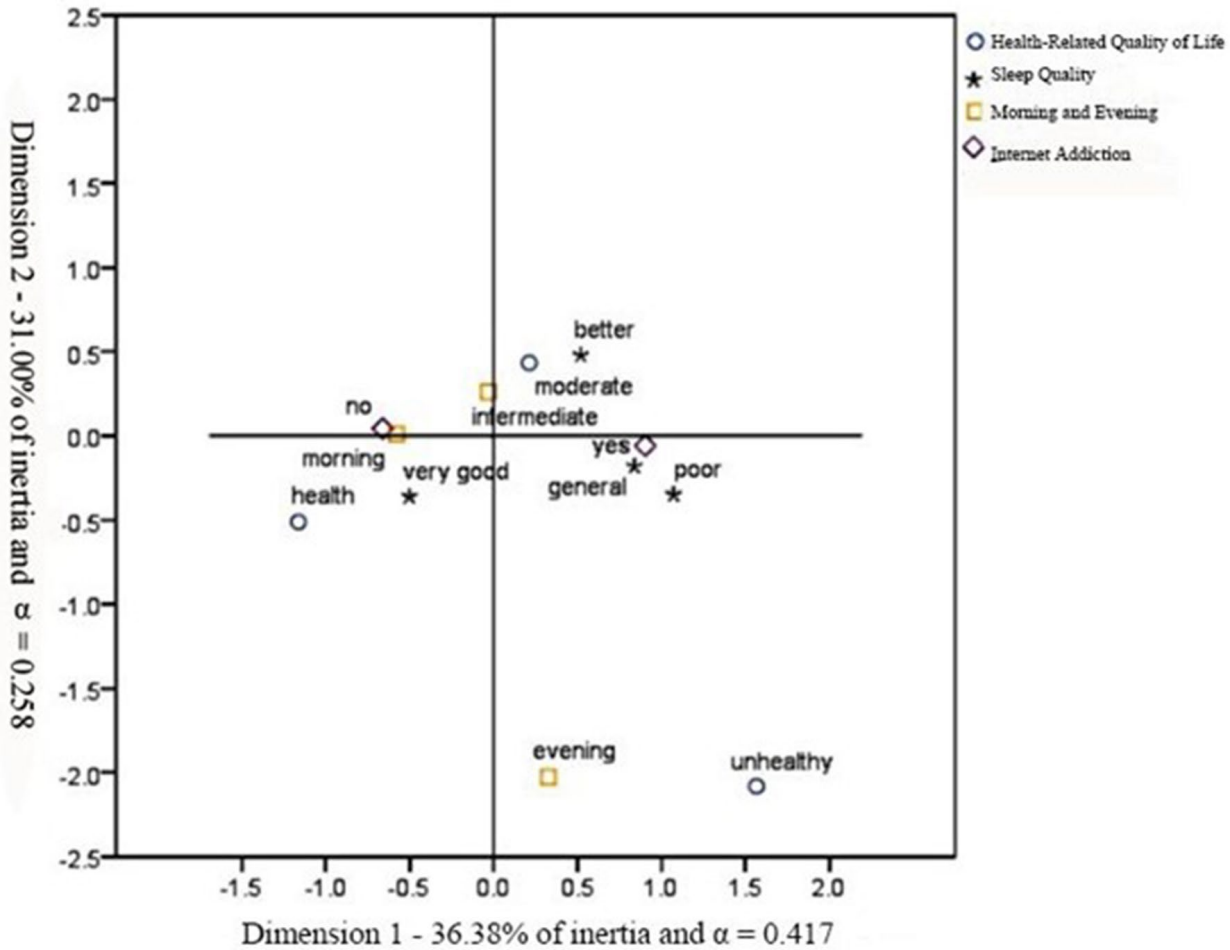
Multiple correspondence analyses of variables related to medical students’ health-related quality of life, sleep quality, morning and evening type, and internet addiction.

A graphical representation of lifestyle associations was provided by MCA (Fig. 1). A total of 67.38% of the variability was explained by dimensions 1 and 2. The internal correlation coefficients (Cronbach’s alpha) were 0.417 and 0.258 respectively, indicating that there is a moderate internal correlation.

The MCA plot showed that individuals with “health quality of life,” “no internet addiction,” “morning type,” and “very good sleep quality” (on the left of the graph) were mostly located opposite individuals with “unhealthy and moderate quality of life,” “internet addiction,” “evening type,” and “poor, general and better sleep quality” (on the right of the graph). Among them, the “very good sleep quality” appeared on the left side alone, while the “poor, general, and better sleep quality” appeared on the right side alone, which indicated that the “very good sleep quality” was quite different from the other three categories. Similarly, the “unhealthy and moderate quality of life” was concentrated on the right, suggesting that people with very good sleep quality would be most likely to have a healthy lifestyle. In addition, individuals with “no internet addiction” and “morning type” were located opposite individuals with “internet addiction” and “evening type”. It can also be noted that there is spatial proximity between “evening type” and “unhealthy quality of life”, which displays a close correlation. In short, MCA analysis was able to differentiate the health-related quality of life, sleep quality, morning and evening type and internet addiction.

### LPA

The fit indices of the LPA models showed that the three-profile model had a lower AIC^40^, BIC^41^, and aBIC^37^. The four-profile model entropy was better than the three-profile model entropy. However, the Lo-Mendel-Rubin likelihood and Vuong-Lo-Mendell-Rubin values for the four-profile model were not significant^37,38^. Considering the above, the three-profile model was chosen as our default, no-covariates latent profile model since it had overall superior metrics compared to alternative models employing other manifest variables and numbers of latent profiles, as well as enabling better profile interpretation (see Appendix 1).

**Figure 2:**
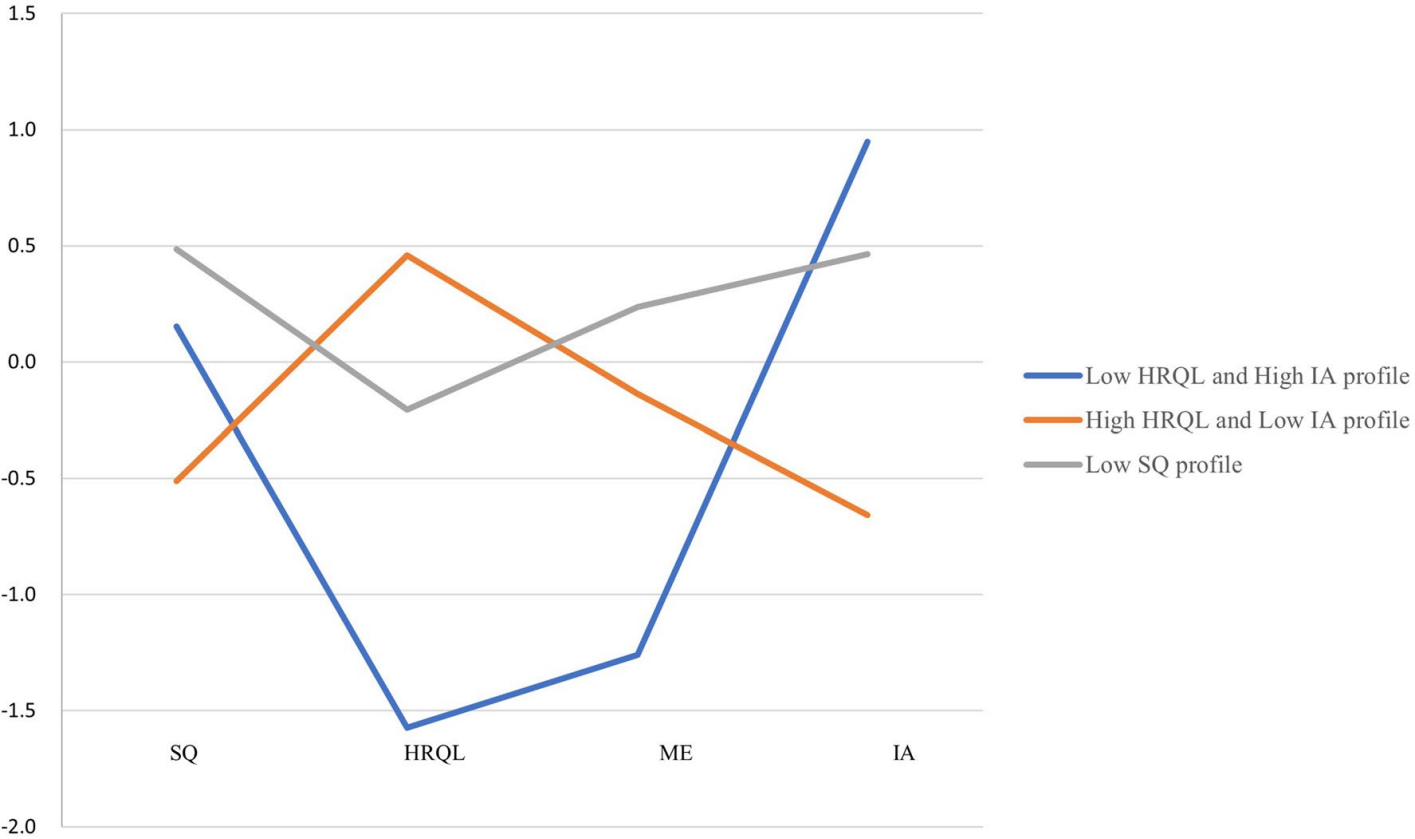
Differences in health-related quality of life, sleep quality, morning and evening type, and internet addiction among medical students for the three profiles. Abbreviations: HRQL, standardized values of health-related quality of life; IA, standardized values of internet addiction; ME, standardized values of morning and evening type; SQ, standardized values of sleep quality. Standardization is to convert the original values of a column of variables into standard scores, and the formula is (original value—mean value of variables)/standard error. After standardization, data with 0 as the average and 1 as the standard deviation are obtained.

Three profiles were named based on their most prominent characteristics and included the Low sleep quality profile, the High health-related quality of life and Low internet addiction profile, and the Low health-related quality of life and High internet addiction profile (Fig. 2). The largest profile (n = 579; 47.42%), which we named the Low sleep quality profile, was characterized by the poorest sleep quality and highest morning and evening type scores that indicated a tendency towards morningness among the three existing profiles. The second-largest profile (n = 499; 40.87%), which we named the High health-related quality of life and Low internet addiction profile, was characterized by the highest level of health-related quality of life but the lowest level of internet addiction. The smallest profile (n = 143; 11.71%), which we named the Low health-related quality of life and High internet addiction profile, was characterized by the highest standardized values of internet addiction but the lowest standardized values of health-related quality of life; this profile of respondents also scored the lowest in the morning and evening type that indicated the greatest eveningness preference among the three existing profiles.

To determine whether the three profiles differed significantly, Kruskal–Wallis tests were carried out (Table 2). The results showed that health-related quality of life, sleep quality, morning and evening type, and internet addiction had significant differences among the three profiles (*P* < 0.001). Especially, the students in profile II scored significantly higher in health-related quality of life than the other two groups, while the scores in sleep quality and internet addiction were significantly lower than those of the other groups. Besides, the students in profile III scored significantly lower in terms of health-related quality of life and morning and evening types than the other two profiles.

**Table 2:** Health-related quality of life, sleep quality, morning and evening type, internet addiction in three profiles.

Items	Median (interquartile)			U value	*p* value	Post hoc test between profiles
	Low sleep quality profile (profile I, n = 579)	High health-related quality of life and Low internet addiction profile (profile II, n = 499)	Low health-related quality of life and High internet addiction profile (profile III, n = 143)			
Health-related quality of life	139.00 (128.00–151.00)	156.00 (143.00–175.00)	104.00 (104.00–131.00)	367.078	< 0.001	Profile I > profile III (*p* < 0.001) Profile II > profile I (*p* < 0.001) Profile II > profile III (*p* < 0.001)
Sleep quality	7.00 (5.00–9.00)	4.00 (2.00–5.00)	6.00 (4.00–8.00)	327.221	< 0.001	Profile I > profile II (*p* < 0.001) Profile I > profile III (*p* < 0.001) Profile II < profile III (*p* < 0.001)
Morning and evening type	49.00 (46.00–52.00)	47.00 (44.00–50.00)	41.00 (38.00–50.00)	101.115	< 0.001	Profile I > profile II (*p* < 0.001) Profile I > profile III (*p* < 0.001) Profile II > profile III (*p* < 0.001)
Internet addiction	54.00 (47.00–60.00)	40.00 (33.00–46.00)	60.00 (54.00–60.00)	530.086	< 0.001	Profile I > profile II (*p* < 0.001) Profile II< profile III (*p* < 0.001) Profile II < profile III (*p* < 0.001)

## Discussion

First, this study explored the profiles of health-related quality of life, sleep quality, morning and evening type, and internet addiction in medical students. LPA identified three profiles (the Low sleep quality profile, the High health-related quality of life and Low internet addiction profile, and the Low health-related quality of life and High internet addiction profile) in this population. Second, these profiles demonstrated differential associations between health-related quality of life, sleep quality, morning and evening type, and internet addiction of medical students, documenting that sleep quality is not only associated with unhealthy lifestyles or morning and evening types, but also related to internet addiction.

Our results suggest that significant differences were observed between males and females in sleep quality, and morning and evening type. Therein, male medical students have poorer sleep quality than female medical students. Both males and females are troubled by changes in their emotional and social environments during their college years^42^. A study showed that the abnormal sleep state of students is mainly affected by the pressure of social interaction^43^. This was particularly true during the COVID-19 pandemic when most students were subjected to closed management on campus and heightened anxiety^44^. Female college students often have more emotional support than male students, and they can often face and solve the problems they encounter through discussions among their partners. Male students are likely to be infected with the bad habit of playing games all night, which may reduce the quality of sleep^45–47^. In addition, from the perspective of sleep conditions, men are more likely to go to bed later than women, resulting in decreased total sleep time^48^.

A combination of these properties is more likely to be associated with unhealthy lifestyles according to the MCA: staying up and waking up later, and poor sleep quality. In contrast, healthy lifestyles are more likely to be accompanied by very good sleep quality, which was consistent with the overall observation. On the one hand, the heavy schoolwork burden, numerous subjects and fierce competition among peers make medical students have strong learning motivation, which forces them to spend more rest time to complete academic tasks and sleep late^49,50^. On the other hand, although the internet provided students with good learning, communication and entertainment platforms during the recent epidemic, it also had a subtle impact on their lifestyles and behaviors^51^. In the face of huge learning pressure and potential interpersonal barriers, medical students are more inclined to take online communication as a way to lessen pressure and obtain a sense of identity and belonging from the virtual media platform^52,53^. Unrestrained Internet surfing will lead to high brain excitement, affect sleep time and sleep efficiency, cause serious sleep disorders and have a negative impact on daytime learning and life^54^. However, medical students’own health literacy and awareness of physical health care are higher than those of the general population, which makes some medical students highly self-disciplined, that is, they have very good sleep quality under a healthy lifestyle to reduce the adverse effects caused by occupational exposure or learning pressure^55,56^. A study showed that a brief and personalized online sleep education can help college students develop good sleep habits, thereby significantly improving their sleep quality^57^. Tsai et al. pointed out in a review article that cognitive behavioral therapy for insomnia has a large effect in the sleep quality improvement among young insomniacs, which may be an effective and inexpensive measure to address sleep deprivation and poor sleep habits among college students^58^. Therefore, the above methods might be applied to improve the sleep quality of medical students.

The LPA data stressed three latent profiles of medical students’ health-related quality of life, sleep quality, morning and evening type, and internet addiction. The emergence of these distinct profiles suggests that medical students’ health does consistently correlate with their lifestyles. For example, in the Low health-related quality of life and High internet addiction profile, students experienced a relatively low level of health-related quality of life but a high degree of internet addiction. Such students’ learning motivation may be weakened due to internet addiction, so they are more vulnerable to academic setbacks^59^. They may feel more anxious and nervous when facing academic and employment pressures unique to medical students, thus affecting sleep time and sleep efficiency, and possibly leading to serious sleep disorders^60^. They may also shrink back when facing challenges in clinical practice and communicating with teachers and their families, thus spending more time on online entertainment, such as web chat, online shopping, watching videos, etc^61^.

Students with high health-related quality of life and low internet addiction profile had the best sleep quality. Our results concerning the link between sleep quality and internet addiction and health-related quality of life have already been identified in previous research^22^. A study on the LPA of sleep quality among university students suggests that students exhibit the worse health-related quality of life in the profile with the poorest perceived sleep quality^62^. Similarly, a study on medical students using a LPA also indicated that a healthy lifestyle is associated with better sleep quality^63^. One possible explanation is that students with high health-related quality of life may be more likely to maintain positive emotions and attitudes, which can contribute to better sleep quality^64^. They may also prioritize social interaction, leading to a lower degree of internet addiction^65^. In addition, students who had high health-related quality of life may be more focused on healthy lifestyle habits, such as the morning types that involve early to bed and early to get up^66^. And students with low internet addiction intent to have a good sleep, for example, they can fall asleep easily and achieves better sleep quality by avoiding the use of electronic devices before bedtime^67,68^. These findings suggest the importance of maintaining a healthy lifestyle, managing internet use, and prioritizing morning types for better sleep quality.

We found that medical students in the Low sleep quality profile had the poorest sleep quality and a tendency towards morningness among the three profiles. This result is contrary to previous research^69^. This could be because students who wake up early tend to have a more regular biological clock and daily routine, making it easier for them to maintain good sleep habits^70^. As a result, students with poor sleep quality are more likely to prefer early rising as a way to improve their sleep quality^71^. According to previous research, people with poor sleep quality may experience shallower sleep, more frequent awakenings, or waking up early^72^. Our findings suggest that maintaining a regular sleep schedule and routine may be beneficial for improving sleep quality. It is important to note that poor sleep quality can have various negative consequences, such as waking up early, which can be improved with good sleep habits.

This study identified health-related quality of life, sleep quality, morning and evening type, and internet addiction in the student population, which may help reduce the negative impact of health-related quality of life, sleep quality, morning and evening type, and internet addiction, improving the efficiency of medical students. Moreover, at present, programmatic attention is largely focused on the general public, with less emphasis on medical students^73,74^. It is of great significance to study the health-related quality of life, sleep quality, morning and evening type, and internet addiction among medical students to improve healthcare service quality in the health system^75^.

Our study has the limitation of recruiting students from only one college. College-specific factors, such as the learning environment and course structure, may affect the results. Thus, international multicenter studies on a larger scale are required to verify the findings described herein in the future.

## Conclusion

Poor sleep quality, evening type, internet addiction, and unhealthy quality of life are risk factors for the health of medical students. Classifying medical students can help predict their health and provide a basis for helping their medical universities and hospitals make targeted policies based on distinctive characteristics.

Although medical students already have some knowledge on health and medicine, they should still need to pay attention to their health, especially at work and at rest. Medical students need to be aware of the existence of these risk factors and take proactive steps to improve their lifestyles and keep healthy. Future guidance includes applying sleep-related health education and behavioural interventions to promote healthy sleep habits, such as establishing a regular sleep schedule, creating a sleep-conducive environment, and avoiding excessive internet access before bedtime. This will help promote a healthy workforce in the foreseeable future. Developing an evidence-based health model for medical students would serve as an essential part of designing and evaluating health promotion measures for medical students.

## Supplementary Information

Supplementary Information.

## Data Availability

The datasets used during the current study are available from the corresponding author on reasonable request.
