# Supplementary material for: Latent profile analysis for health-related quality of life, sleep quality, morning and evening type, and internet addiction among medical students
Source: Sci Rep. 2023 Jul 12;13:11247. doi: 10.1038/s41598-023-38302-7 (PMC10338532; doi:10.1038/s41598-023-38302-7)
Supplement: Supplementary file 1 — Supplementary Information. [file 41598_2023_38302_MOESM1_ESM.pdf]

Latent profile analysis for health-related quality of life, sleep quality, morning and evening type, and internet addiction among medical students

Author information:

Ling Yu[2#], B.S. ; Yifan Wu[1, 3#], B.S. ; Chaowei Guo[1#], B.S. ; Qiao Qiao[4] Ph.D. ; Xue Wang[1], B.S. ; Shuang Zang[1]*, M.D.

[#] These authors share the first authorship on this work.

Author affiliations:

[1] Department of Community Nursing, School of Nursing, China Medical University

[2] Phase I Clinical Trails Center, The First Hospital of China Medical University

[3] School of Nursing, Jilin University

[4] Department of Radiation Oncology, The First Hospital of China Medical University

Ling Yu, B.S., Phase I Clinical Trails Center, The First Hospital of China Medical University.

Mail address: No.155 Nanjing Bei Street, Heping District, Shenyang, Liaoning Province, 110001, China

E-mail: yl_cmu@126.com

Yifan Wu, B.S.,

Department of Community Nursing, School of Nursing, China Medical University.

Mail address: No.77 Puhe Road, Shenyang North New Area, Shenyang, Liaoning Province, 110122, China

School of Nursing, Jilin University.

Mail address: 965 Xinjiang Street, Changchun, Jilin Province, 130021, China

E-mail addresses: 15142990805@163.com

Chaowei Guo, B.S., Department of Community Nursing, School of Nursing, China Medical University.

Mail address: No.77 Puhe Road, Shenyang North New Area, Shenyang, Liaoning Province, 110122, China

E-mail: gcw18830901603@126.com

Qiao Qiao, Ph.D., Professor, Department of Radiation Oncology, The First Hospital of China Medical University.

Mail address: No.155 Nanjing North Street, Heping District, Shenyang, Liaoning Province, 110001, China

E-mail: braveheart8063@outlook.com

Xue Wang, B.S., Department of Community Nursing, School of Nursing, China Medical University.

Mail address: No.77 Puhe Road, Shenyang North New Area, Shenyang, Liaoning Province, 110122, China

E-mail: 13909822541@163.com

***Corresponding author:**

Shuang Zang*, M.D, Associate professor, Department of Community Nursing, School of Nursing, China Medical University.

Mail address: No.77 Puhe Road, Shenyang North New Area, Shenyang, Liaoning Province, 110122, China

E-mail: zangshuang@cmu.edu.cn

Tel +86 18900910689

Fax +86 02431939546

Appendix 1 Fit statistics for the latent profile analysis.

Number of Profiles	AIC	BIC	aBIC	VLMR-LRT	LMR
2	6673.808	6760.634	6706.635	<0.001	<0.001
3	6638.909	6771.702	6689.116	<0.001	<0.001
4	6640.116	6818.876	6707.701	0.192	0.198

Note. “AIC” = Akaike information criterion. “BIC” = Bayesian information criterion. “aBIC” = Sample size-adjusted Bayesian information criterion. “VLMR-LRT” = Vuong-Lo-Mendell-Rubin likelihood ratio test. “VLRT” = Lo-Mendell-Rubin Adjusted LRT Test.
